# Mismatch Negativity Latency as a Biomarker of Amnestic Mild Cognitive Impairment in Chinese Rural Elders

**DOI:** 10.3389/fnagi.2015.00022

**Published:** 2015-03-12

**Authors:** Li-Li Ji, Yuan-Yuan Zhang, Lane Zhang, Bing He, Guo-Hua Lu

**Affiliations:** ^1^Department of Medical Nursing, Weifang Medical University, Wei Fang, China

**Keywords:** amnestic mild cognitive impairment, Alzheimer’s disease, event-related potentials, mismatch negativity, biomarkers

## Abstract

The aim was to evaluate the mismatch negativity (MMN) component, a correlate of the automatic detection of changes in the acoustic environment, in healthy adults, and adults with amnestic mild cognitive impairment (aMCI). Forty-three aMCI subjects and 43 healthy Chinese older adults were arranged into experimental group and control group, respectively. Their MMN amplitude and latency were measured at the FZ, FCZ, and CZ electrode sites under a passive auditory oddball task. The results showed that the latencies obtained from the FZ, FCZ, and CZ electrode sites were significantly longer in the aMCI adults than in the control adults (*P * < 0.01) while there were no significant differences in MMN amplitude between two groups (*P * >* *0.05). The MMN latency was found to be a sensitive and specific biomarker of aMCI.

## Introduction

Mild cognitive impairment (MCI) refers to the gray zone between the cognitive changes of normal aging and very early dementia (Grundman et al., 2004a; Stephan et al., 2013). Individuals with MCI show cognitive impairment greater than expected for their age, but otherwise are functioning independently and do not meet the commonly accepted criteria for dementia (Petersen et al., 1999).

The incidence of MCI ranges from 1 to 6% per year while prevalence estimates range from 3 to 22% per year in western developed countries (Hanninen et al., 2002; Larrieu et al., 2002; Ganguli et al., 2004; Roberts et al., 2012). Recently, one large cross-sectional study (Jia et al., 2014) conducted in China, using a multistage cluster sampling design, included a total of 10,276 community residents (6096 urban, 4180 rural) aged 65 years or older, found a prevalence rate of 20.8% for MCI in Chinese elders. The study also stated that rural population had a higher prevalence of overall MCI than urban population (23.4 vs. 16.8%). CRCA (China Research Center on Aging, 2014) has estimated that 1 of every 9 persons is 65 years old or over, totaling 119 million people at the end of 2010 in China. By the year 2050, those numbers are projected to double with the actual number of people over age 65 projected to be almost 300 million, at which point the population of older persons will be accounted for almost 22% of the Chinese population (2014). As the high prevalence of MCI in Chinese rural elders and the rate at which the Chinese population is aging has accelerated, we can forecast that there would be a huge population (about 35.1 million) of elders with MCI by the year 2050 in the rural of China. However, with the progressive urbanization of china, more medical support will be supplied for urban meanwhile medical resources will be even more sparse in rural areas. As a result, the MCI patients in rural areas can hardly get medical help.

Among the different subtypes of MCI, amnestic mild cognitive impairment (aMCI) is the most likely to progress to Alzheimer’s disease (AD) (Albert et al., 2011), which is the most prevalent form of dementia in the elderly (Papaliagkas et al., 2009). Subjects with aMCI develop dementia at 10–15% per year (Grundman et al., 2004b; Misra et al., 2009) as compared to the general population of 1–2% (Bischkopf et al., 2002). Delaying or preventing the onset of dementia by a mere 1 year alone could translate into one million fewer number of cases than predicted by the year 2050 (Brookmeyer et al., 1998).

Thus, Biomarkers are needed to facilitate early identification of aMCI and predict progression to dementia. The methods used to search for biomarkers of aMCI include scales (Duchesne et al., 2005; Hoops et al., 2009; Kasten et al., 2010), neuroimaging techniques (Small et al., 2006; Hamalainen et al., 2007), cerebrospinal fluid analysis (Perneczky et al., 2011), and genetic analysis (Zhang et al., 2012), which are invasive or expensive or requires a high level of education, could not be widely used in rural population. Hence, the identification of objective biomarkers that are sensitive to the pathophysiological changes in aMCI and easily accepted in rural population is important for both prevention of dementia and promotion of health.

Mismatch negativity (MMN) relates to the difference wave obtained by subtracting the standard stimulus ERP from the deviant stimulus ERP and usually peaks between 150 and 250 ms after presentation of the deviant stimulus (Nagai et al., 2013). On electroencephalogram (EEG), maximal MMN responses are evident at front central scalp recording sites, with phase reversal at mastoids (Nagai et al., 2013).

Whether MMN technique would be such aMCI biomarkers warrant consideration founded on three essential characteristics designated as ideal (Lindin et al., 2013): it is non-invasive, simple to measure, and inexpensive. Moreover, MMN is even elicited under passive conditions when subjects ignore the stimuli, which means it does not need the cooperation of participants and it would not be limited by educational level of participants.

Previous studies have shown that MMN is a promising biomarker candidate for cognitive impairment in Parkinson’s disease (Cai et al., 2004), brain trauma (Wijnen et al., 2007), Alzheimer disease (Tales et al., 2008), and schizophrenia (Umbricht and Krljes, 2005). In these reported researches, the MMN latencies and (or) amplitude were significantly different between experimental group and control group. Despite the increasing number of research about MMN in other diseases, there are only two published studies to date evaluating the effect of MCI on MMN parameters. Mowszowski et al. (2012) recorded ERPs in an Australia sample of 14 healthy adults and 28 adults with MCI, in a passive oddball task in which the standard and deviant stimuli differed in duration (standard: 50 ms, deviant: 100 ms). They did observe that at mastoid locations, the MMN amplitude was smaller in the MCI group than in the control group, which the authors considered reflect of the inefficiency of processing information in an early pre-attentional stage in the MCI group. Lindin et al. (2013) studied MMN component in Spain healthy adults and adults with aMCI (age range: 50–87 years) using auditory–visual attention–distraction task and found that MMN amplitude at the Cz electrode site was significantly smaller in the aMCI adults than in the control adults, suggesting MMN to be a sensitive and specific biomarker of aMCI.

The recent two studies provided some interesting results, but also presented some limitations. Thus, the former study obtained MMN amplitude at mastoid electrodes, but not at the frontocentral locations, where MMN is typically identified and analyzed (Mowszowski et al., 2012); although the latter study obtained MMN at the frontocentral locations, they only evaluated it at the CZ electrode site, and did not take into account the possible effects of interactions between the electrode sites (FZ, FCZ, CZ) and Group factors (Lindin et al., 2013). Moreover, these two studies were carried out in Australia and Spain, respectively; further researches would be needed in Chinese population.

The aims of the present study were as follows: (1) to determine any differences in MMN parameters between healthy adults and adults with aMCI in Chinese rural population; (2) to evaluate whether such differences between healthy adults and adults with aMCI are affected by electrode sites, by considering three electrode sites (FZ, FCZ, CZ); (3) to evaluate whether MMN changes associated with aMCI are sensitive and specific biomarkers of this syndrome.

## Materials and Methods

### Participants

Forty-three aMCI subjects and 43 healthy Chinese older adults selected from rural villages of Weifang, Shandong, China were arranged into experimental group and control group, respectively.

Experimental group: there were 22 male and 21 female subjects in this group, the average age was 65.81 ± 6.90 years and the average years of education were 3.88 ± 2.80. The participants were selected according to the U.S. mental disorders’ fourth edition of the Diagnostic and Statistical Manual (DSM-IV) (Association and DSM-IV, 1994) in mild neurocognitive damage standards and the Diagnosis standard of Shanghai Mental Health Center (Shi Fu and Wei, 1999): (1) memory complaints corroborated by an informant; (2) Mini-Mental State Examination (MMSE) Score ≤26 points, the level of Global Deterioration Scale (GDS) assessment is between 2 and 3; (3) activity of daily living scale (ADL) Score ≤18 points; (4) Hachinski ischemia index (HIS) ≤4 points; (5) course of cognitive impairment >3 months; (7) Does not meet DSM-IV (1) criteria for dementia syndrome.

Control group: there were 19 male and 24 female subjects in this group, the average age was 66.21 ± 6.81 years and the average years of education were 4.90 ± 2.76. The participants were selected according to the following criteria: (1) no memory complaint; (2) MMSE total score ≥24; (3) intact instrumental activities of daily living.

Subjects with any of the following condition were excluded from the study: (1) left-handed; (2) visual or hearing difficulty; (3) depression; (4) history of head trauma; (5) heart, lung, liver, or kidney failure; (6) active neurological or psychiatric conditions; (7) any severe illness that may affect cognitive function; (8) drug or alcohol abuse.

Ethical approval for this study was obtained from the Research Ethics Committee, Weifang Medical University, and written informed consents were obtained from all of the subjects.

There were no significant differences in gender, age, or educational level between the experimental and control groups as shown in Table 1.

**Table 1:** **Characteristics of the study sample (aMCI vs. HC)**.

Variable	aMCI	HC	*P*
Age	65.81 ± 6.90	66.21 ± 6.81	0.787
Female	21 (49%)	24 (56%)	0.517
Education (years)	3.88 ± 2.80	4.90 ± 2.76	0.093

*aMCI, amnestic mild cognitive impairment; HC, healthy controls*.

### Stimuli and task

A passive auditory oddball task was used in this study. Participants were presented with 500 auditory stimuli (divided in two blocks separated by a 2-min rest interval). Auditory stimuli were sounds, presented binaurally via headphones. Two kinds of sounds were presented: 85% were standard stimuli (tone bursts, 1000 Hz, 85 dB) and 15% were deviant stimuli (tone bursts, 2000 Hz, 90 dB).

### Electroencephalographic recording

The participants were seated on a comfortable chair in a Faraday chamber, with attenuated levels of light and noise, and were instructed to move as little as possible during the recording. The EEG was recorded from 64 ring electrodes placed in an elastic cap, according to the International 10–20 system. All electrodes were referenced to an electrode attached to the tip of the nose, and an electrode positioned at Fpz served as ground. The vertical electro-oculogram (EOG) was recorded from two electrodes placed supra and infra-orbitally on the left eye, and the horizontal EOG was recorded from two electrodes placed at the outer canthi of both eyes. The EEG was continuously digitized at a rate of 1000 Hz (band pass 0.05–100 Hz), and electrode impedances were kept below 5 kΩ. Finally, to identify and measure MMN, we obtained the deviant minus standard (D − S) difference waveforms. The MMN component was identified as a negative wave in the 100–250 ms interval, and it was evaluated at the FZ, FCZ, and CZ electrode sites, respectively. The MMN amplitude (in microvolts, from the maximum peak to the baseline) and latency (in milliseconds, from the auditory stimulus onset to the maximum peak) were measured.

### Statistical analysis

Repeated measure analysis of variance (ANOVA) was conducted to investigate the effect of the Group (Experimental group, Control group) and Electrode Sites (FZ, FCZ, CZ) factors on the MMN amplitude and latency. The comparison on various characteristics was conducted using Student’s test (for continuous variables) or chi-square test (for dichotomous variables). All statistical analysis was performed using SPSS 18.0. Differences were considered significant at *p* < 0.05.

## Results

### MMN latency

**Figure 1:**
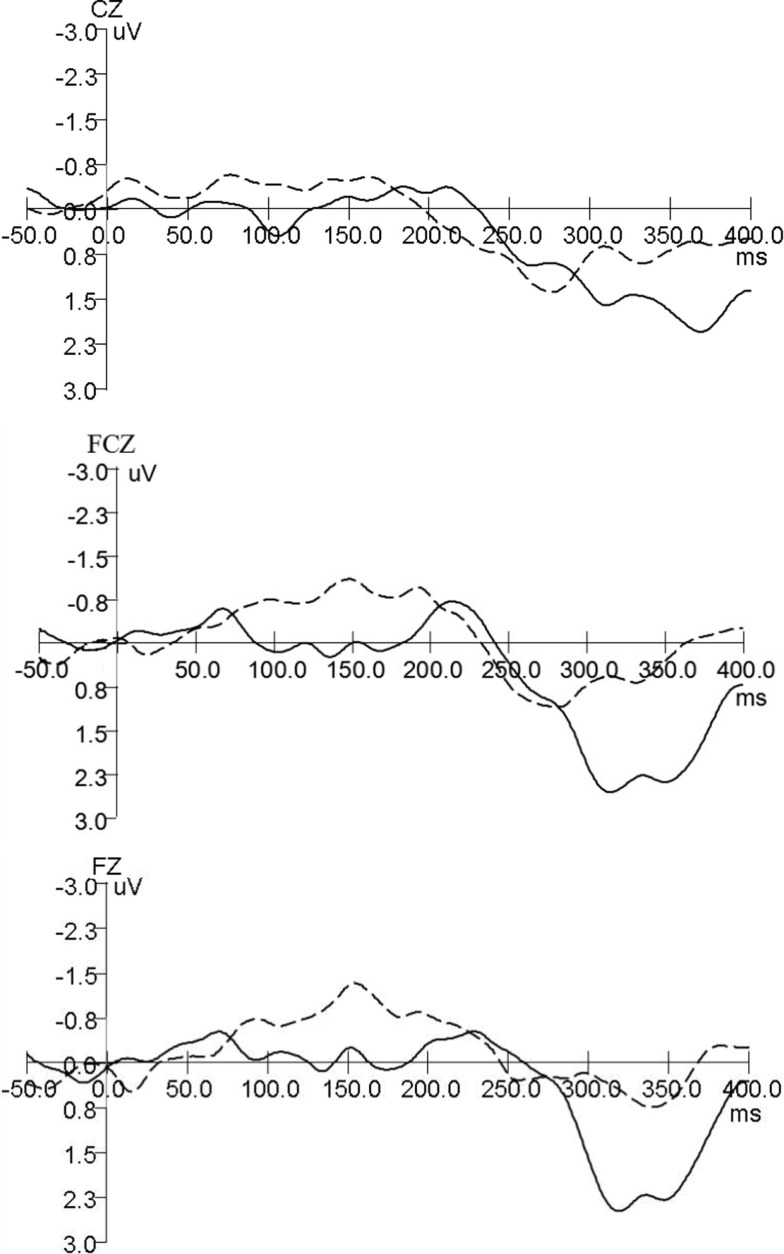
**Grand average MMN wave forms for aMCI and healthy controls**. Mismatch negativity (MMN) component was identified in both groups of participants (control and aMCI). Control and aMCI adults showed differences for MMN component (— aMCI, … healthy controls).

The grand average MMN wave forms for aMCI and healthy controls are shown in Figure 1, healthy adults in the current study have a negative-going MMN in the 100–250 ms time window at the FZ and FCZ electrode while aMCI patients showed no clear MMN. For MMN latency, repeated ANOVA (Group × Electrode Sites) showed significant effects of the Electrode Sites factor (*F* = 61.984, *P* < 0.001), the Group factor (*F* = 197.573, *P* < 0.001), and the Group × Electrode Sites interaction (*F* = 11.728, *P* < 0.001) (Table 2). The latencies obtained from the FZ, FCZ, and CZ electrode sites were significantly longer in the MCI group than in the control group (*P * < 0.05) (Table 3). For the aMCI group, FZ latency > FCZ latency > CZ latency (*P* < 0.01); for the control group, FZ latency > CZ latency (*P* < 0.01) and FCZ latency > CZ latency (*P* < 0.01), but there was no significant difference between FZ latency and FCZ latency (*P * > 0.05) (Table 3).

**Table 2:** **The effects of electrode location and group on ERP amplitude and latency**.

Variable	Electrode site	Group	Location × group
MMN amplitude	25.770***	0.688	2.586
MMN latency	61.984***	197.573***	11.728***

*This is the main effect (electrode site, group) and interaction effect (electrode site × group) using a two-factor analysis of variance*.

*Electrode site: FZ, FCZ, CZ; Group: MCI vs. HC*.

***Indicates *p* < 0.001.

**Table 3:** **Comparison of MMN latency between aMCI and healthy controls**.

Electrode	Latency		
	aMCI	Controls	*P*
FZ	237.47 ± 9.145	197.44 ± 14.730	<0.001
FCZ	227.16 ± 9.947	196.56 ± 13.191	<0.001
CZ	214.21 ± 17.246	188.26 ± 13.510	<0.001

*This is the *post hoc* test results of the mean values of latencies between aMCI and control groups*.

### MMN amplitude

Repeated ANOVA (Group × Electrode Sites) showed significant effects of the Electrode Sites factor (*F* = 25.770, *P* < 0.001), but there was no significant Group factor (*F* = 0.688, *P * >* *0.05) or Group × Electrode Sites interaction (*F* = 2.586, *P* > 0.05) (Table 2).

## Discussion

Mismatch negativity component was identified in both groups of participants (control and aMCI). Control and aMCI adults showed differences for MMN component, healthy adults in the current study have a negative-going MMN with an evident peak in the 100–250 ms time window at the FZ electrode while aMCI patients showed no clear MMN. However, when compared to the clear MMN peak at −4 μV at Cz in Lindin et al. (2013), the MMN here in the healthy group seems to be not that prominent (−1.5 μV). The reason might be the sample differences of this study and Mónica Lindínl’s. The average age of healthy adults in the current study was 66.21 ± 6.81, while the clear MMN peak at −4 μV in Lindin et al. were from the middle-aged subgroup (50–64 years), the MMN from the older age subgroup (65 years and over) was not that prominent either in Mónica Lindín et al.

The MMN latencies obtained from all three electrode sites (FZ, FCZ, and CZ) were significantly longer in the aMCI group than in the CG, and the difference is more obvious at the FZ electrode site. This result is intriguing because different results about MMN latencies were reported in the only two previous studies comparing MCI group with a control group. Lindin et al. (2013) reported that the MMN latency was significantly shorter in the aMCI group than in the CG. Mowszowski et al. (2012) also observed slightly shorter (but non-significant) MMN latencies (measured at Fz and Cz electrodes) in the MCI than in the control group. Despite the controversy with the two previous studies, the result of the present study on MMN latency is consistent with previous studies comparing other diseases that may cause cognitive impairment with a control group. Cai et al. (2004) evaluated MCI in Parkinson’s disease with MMN and found that the peak latency of MMN in the PD group was significantly longer than in the control group. Kathmann et al. (1995) reported delayed MMN peak latencies in chronic alcoholics, in comparison with their healthy peers. Lou et al. (2006) evaluated MMN of patients with first-episode schizophrenia and reported delayed MMN latency in schizophrenics compared to healthy controls, similar results were found in another research in chronic soldiers’ schizophrenics (Gao et al., 2007). Moreover, prolonged latencies were reported in experimental group in the clinical researches of MMN in patients with AD (Chen et al., 2003).

Inconsistent with the two previous studies comparing MCI group with a control group, no significant group factor was found in MMN amplitude in the present study. Mowszowski et al. (2012) observed a larger MMN amplitude in healthy adults than in a multi-domain MCI group (for an age range of 50–90 years in both groups). Mónica Lindín et al. found the MMN amplitude was significantly larger in the CG than the aMCI group, only for the middle-aged subgroup (50–64 years), but not for adults 65 years old or more. The authors tentatively speculate that this is probably due to a significant age-related decrease in MMN amplitude in the CG, as also found by Gaeta et al. (1998). The lack of differences between the CG and the aMCI group may be due to an age-related decline in the mechanism for echoic memory trace maintenance and/or the pre-attentional mechanisms involved in the automatic detection of differences in the acoustic environment, which may mask the effects of aMCI on that parameter (Lindin et al., 2013). This might also be the reason of the present study of no significant group factor in MMN amplitude. Further studies are needed to identify this.

As found in the present study, MMN latencies were significantly longer in aMCI adults, the MMN latency may be considered a biomarker of aMCI. Moreover, the characteristics of the MMN component make it an ideal biomarker; it is an automatic ERP component, which is not dependent on the attention given by the subject to the task and, moreover, it is obtained in a non-invasive manner and is simple and inexpensive to measure. However, in consideration of the discordance between our findings and the two previous studies, which reported earlier (latency) and/or smaller (amplitude) MMNs in the aMCI population (Mowszowski et al., 2012; Lindin et al., 2013), both contrary to the findings of our study, a more in-depth approach to potential ways to address this discordance in future work would be needed to clarify whether MMN latency can be a fairly sensitive and specific psychophysiological biomarker for the identification of adults with aMCI.

This study is not without limitations that mainly include unknown group differences in risk factors of cognitive decline and AD, such as apolipoprotein E e4 genotype, and inflammation, although there were no significant differences between groups in terms of gender, age, or educational level. Besides, in consideration of the relatively small sample size of this study, reference values of MMN latency and amplitude for both aMCI and healthy elders could not be offered. Future studies, with a large sample of participants, should determine reference values of MMN latency and amplitude for aMCI elders.

## Conflict of Interest Statement

The authors declare that the research was conducted in the absence of any commercial or financial relationships that could be construed as a potential conflict of interest.
